# The Impact of a Structured Contraceptive Counseling Program on Reproductive Health Knowledge, Sexual Autonomy, and Mental Well-Being Among Romanian College Women

**DOI:** 10.3390/healthcare13080955

**Published:** 2025-04-21

**Authors:** Denisa Hinoveanu, Adrian Gluhovschi, Ileana Enatescu, Oana Belei, Lavinia Stelea, Catalin Dumitru, Felix Bratosin, Sorina Maria Denisa Laitin

**Affiliations:** 1 Department of Obstetrics and Gynecology, “Victor Babes” University of Medicine and Pharmacy Timisoara, 300041 Timisoara, Romania; adriana.hinoveanu@umft.ro (D.H.); gluhovschi.adrian@umft.ro (A.G.); stelea.lavinia@umft.ro (L.S.); dumitru.catalin@umft.ro (C.D.); 2 Doctoral School, “Victor Babes” University of Medicine and Pharmacy Timisoara, Eftimie Murgu Square 2, 300041 Timisoara, Romania; 3 Discipline of Neonatology, “Victor Babes” University of Medicine and Pharmacy Timisoara, 300041 Timisoara, Romania; 4 First Pediatric Clinic, Disturbances of Growth and Development on Children Research Center, “Victor Babes” University of Medicine and Pharmacy Timisoara, 300041 Timisoara, Romania; 5 Department of Infectious Disease, “Victor Babes” University of Medicine and Pharmacy Timisoara, 300041 Timisoara, Romania; felix.bratosin@umft.ro; 6 Discipline of Epidemiology, “Victor Babes” University of Medicine and Pharmacy Timisoara, 300041 Timisoara, Romania; laitin.sorina@umft.ro

**Keywords:** contraceptive counseling, reproductive health, mental well-being, college women, sexual autonomy, anxiety, depression

## Abstract

Background and Objectives: Contraceptive education may influence reproductive health outcomes, foster greater sexual autonomy, and improve mental well-being. The current study investigated the efficacy of a structured contraceptive counseling program on reproductive health knowledge, sexual autonomy, anxiety, and depressive symptoms among Romanian college women. Materials and Methods: A prospective study was conducted during the 2021–2024 academic years, enrolling 240 female students aged 18–26 from the “Victor Babes” University of Medicine and Pharmacy in Timisoara. The participants were divided into two arms: (1) Intervention Group (IG, n = 115), which was provided counseling materials, and (2) Control Group (CG, n = 116), which received no additional counseling. Baseline and post-intervention data were collected using a contraceptive knowledge quiz (CKQ), a sexual autonomy scale (SAS), the WHOQOL-BREF, GAD-7, and PHQ-9. Results: After the 12-week intervention, the IG demonstrated significant improvements in contraceptive knowledge (mean CKQ score: 25.5 ± 3.1 vs. 20.1 ± 4.3 in CG, *p* < 0.001), sexual autonomy (SAS: 82.6 ± 9.2 vs. 75.7 ± 10.1, *p* < 0.001), and psychological well-being indicators: lower anxiety (GAD-7: 3.1 ± 2.0 vs. 5.2 ± 2.3, *p* < 0.001) and depression scores (PHQ-9: 4.8 ± 2.0 vs. 7.1 ± 2.2, *p* < 0.001). Multiple regression analysis showed that membership in the IG was the most significant predictor of improved post-intervention contraceptive knowledge and reduced mental distress, even after adjusting for confounders such as age, socioeconomic status, and relationship status. Conclusions: A structured contraceptive counseling program can significantly enhance reproductive health knowledge, strengthen sexual autonomy, and reduce depressive and anxiety symptoms among Romanian college women. The findings emphasize the importance of developing systematic, multi-faceted interventions for improving students’ reproductive well-being and mental health outcomes within university settings.

## 1. Introduction

The decision to have children significantly affects various aspects of life, including educational and career opportunities, especially for college students who stand at a critical junction of personal and professional development [1,2]. In Romania, the cultural fabric is steeped in traditional values that significantly influence decisions about family planning and reproductive health. These traditions often emphasize early marriage and the importance of having children, which can lead to a range of pressures on young women to conform to expected life trajectories, impacting their educational and career aspirations [3,4,5]. The socio-political landscape in Romania also plays a crucial role in shaping attitudes towards reproductive health. Although the country has made strides in improving access to healthcare, gaps in reproductive health education persist, particularly in rural areas where traditional values are most influential. This disparity in access and education leads to varying levels of contraceptive knowledge and utilization, which is critical for enabling young women to make informed choices about their reproductive health.

The correlation between contraceptive knowledge and the postponement of childbirth among college students reveals a complex interplay of educational attainment, career planning, and personal health [6,7]. Studies indicate that higher levels of contraceptive knowledge are associated with better planning for future pregnancies, leading to enhanced academic and career outcomes [8,9,10]. However, gaps in this knowledge can lead to increased rates of unplanned pregnancies, which might disrupt educational trajectories and result in significant psychosocial stress.

Mental health disparities between those who plan their pregnancies and those who do not are well-documented. Unplanned pregnancies can lead to increased anxiety, depression, and stress, particularly among young women in academic environments [11,12]. This relationship underscores the importance of robust sexual education programs that not only provide information but also empower women to make informed reproductive choices [13,14].

The social implications of having children while in college also extend to perceptions of quality of life. Those who delay pregnancy often report higher satisfaction in terms of personal development and financial stability [15,16]. Conversely, students who become parents might experience isolation and reduced peer support, factors that could diminish perceived quality of life and mental well-being [17].

Romania’s healthcare system provides contraceptive services and support to young mothers, yet it faces several challenges. These services are available through public hospitals and community health centers, but access varies significantly between urban and rural areas. There are also educational gaps in comprehensive sex education, influencing contraceptive use and awareness among young people. In comparison to other EU countries, Romania struggles with lower contraceptive use and higher teenage pregnancy rates, due in part to cultural conservatism and limited healthcare funding, highlighting the need for targeted improvements in both education and healthcare provision.

Considering these dynamics, this study aims to explore the intricate connections between contraceptive knowledge, the decision to have children, and the subsequent effects on quality of life and mental health among college students in Romania. We hypothesize that increased contraceptive knowledge is positively correlated with better mental health outcomes and higher quality of life. The objectives include (1) assessing the level of contraceptive knowledge among college women in Romania and its impact on sexual autonomy, anxiety and depression, (2) comparing quality of life indices between female students, and (3) analyzing mental health status across these groups using standardized psychological assessment tools.

## 2. Materials and Methods

### 2.1. Research Design and Ethical Considerations

This longitudinal study was conducted at the Victor Babeș University of Medicine and Pharmacy in Timișoara, Romania. Ethical approval was obtained from the university’s ethics committee before the commencement of data collection. Written informed consent was acquired from all participants after explaining the objectives and procedures of the study. Participant confidentiality was maintained by assigning unique identifiers and storing data securely.

### 2.2. Inclusion Criteria

The study included female college students enrolled at the Victor Babeș University of Medicine and Pharmacy during the academic years 2021–2024. A total of 260 female students aged 18–26 were approached for participation. Inclusion criteria were as follows: (1) enrollment in any undergraduate or graduate program at the university; (2) age ≥ 18; (3) self-reported heterosexual activity in the past 12 months. Exclusion criteria comprised the following: (1) self-reported pregnancy at baseline; (2) history of infertility or hysterectomy; (3) current severe psychiatric conditions requiring in-patient care.

After screening, 240 eligible participants were randomly assigned to either the Intervention Group (IG, n = 120) or the Control Group (CG, n = 120) by a computer-generated randomization list. The Intervention Group (IG) received contraceptive counseling materials. Content included the following: comprehensive overview of contraceptive methods; information on local healthcare resources; interactive role-playing for communication with partners; psychosocial support discussions addressing stigma and cultural beliefs. Control Group (CG): No structured counseling sessions or counseling materials were provided.

### 2.3. Variables

Independent variables included contraceptive knowledge and the presence or absence of children. Dependent variables were quality of life and mental health status. Contraceptive knowledge was assessed through a custom survey developed for this study (a 30-item, multiple-choice instrument. Total scores ranged from 0 to 30; higher scores = higher knowledge), while quality of life and mental health were measured using standardized instruments: the SF-36 (Short Form Health Survey) [18], WHOQOL-BREF (World Health Organization Quality of Life-BREF) [19], GAD-7 (General Anxiety Disorder-7) [20], and PHQ-9 (Patient Health Questionnaire-9) [21], and the women’s sexual autonomy [22]. Demographic and background variables such as age, BMI, substance use behavior, place of origin, age of onset of sexual activity, history of sexually transmitted diseases (STDs), and history of abortion were also collected.

### 2.4. Study Instruments

The Short Form (36) Health Survey is a 36-item survey that measures eight domains of health: physical functioning, role limitations due to physical health problems, bodily pain, general health perceptions, vitality, social functioning, role limitations due to emotional problems, and mental health. Each domain is scored from 0 to 100, with higher scores indicating better health status. The overall score is derived by averaging these domains. In previous studies, the SF-36 has demonstrated high reliability, with Cronbach’s alpha values typically exceeding 0.80 for most domains.

The Brief Version of the World Health Organization Quality of Life survey includes 26 items which measure four domains: physical health, psychological health, social relationships, and environment. Each item uses a five-point scale and domain scores are scaled in a positive direction with higher scores denoting higher quality of life. Cronbach’s Alphas for physical health (α = 0.82); psychological (α = 0.81); environment (α = 0.80); social relationships (α = 0.68) [19].

The General Anxiety Disorder 7-item scale was used to assess the severity of generalized anxiety symptoms. Scores range from 0 to 21, with score ranges of 5, 10, and 15 representing mild, moderate, and severe anxiety symptoms, respectively. This tool is highly reliable, with a Cronbach’s alpha generally around 0.92.

The Patient Health Questionnaire 9-item scale is a multipurpose instrument for screening, diagnosing, monitoring, and measuring the severity of depression. Scores also range from 0 to 27, with thresholds set at 5, 10, 15, and 20 representing mild, moderate, moderately severe, and severe depression. The PHQ-9 consistently reports a Cronbach’s alpha of approximately 0.89, indicating high reliability.

We employed a non-probabilistic convenience sampling technique to recruit female college students from the Victor Babes University of Medicine and Pharmacy in Timisoara, Romania, during the academic year 2023–2024. Data were collected through structured interviews administered at designated locations within the university campus, including the central library and student common areas. These interviews were conducted by trained research assistants who received comprehensive training on survey administration, ethical considerations, and confidentiality protocols to ensure consistency and reliability in data collection. Each interview lasted approximately 15–20 min, allowing for thorough yet efficient data gathering.

### 2.5. Statistical Analysis

The sample size was calculated to ensure adequate power to detect significant differences between the two groups. Assuming a medium effect size (Cohen’s d = 0.5), an alpha level of 0.05, and a power of 0.80, a total sample size of approximately 128 participants (64 in each group) was determined necessary using G*Power software v3.1. To account for potential non-response or incomplete data, the sample was increased by 20%, leading to a target sample size of approximately 154 participants (77 in each group).

The assumption for data normality was verified using Shapiro–Wilk tests. Additionally, the homogeneity of variance assumption, which stipulates that the variances among the groups should be equal, was assessed using Levene’s test. Data analysis was performed using SPSS software v.27. Descriptive statistics (mean ± SD) will be calculated for all quantitative variables. Differences between groups were analyzed using independent *t*-tests for continuous variables and Chi-square tests for categorical variables. Analysis of Covariance (ANCOVA) was used for post-test differences, controlling for baseline scores. Pearson correlation and multiple regression analyses were used to examine the relationships between contraceptive knowledge, quality of life, and mental health outcomes. A *p*-value of less than 0.05 was considered statistically significant.

## 3. Results

A total of 240 participants were enrolled at baseline, and 231 completed the full study, with slightly higher attrition noted in the Intervention Group (IG, n = 5) than in the Control Group (CG, n = 4). Baseline characteristics were broadly comparable between groups, indicating successful randomization (Table 1). Neither group showed significant differences in demographic variables—such as age, BMI, or place of origin—nor in lifestyle factors such as smoking and alcohol use. Similarly, baseline reproductive health indicators (e.g., history of STDs) and outcome measures (e.g., CKQ, SAS, WHOQOL-BREF domains, GAD-7, PHQ-9) were statistically equivalent (Table 1 and Table 2), suggesting that any post-intervention effects could be attributed primarily to the structured counseling program rather than pre-existing disparities.

**Figure 1:**
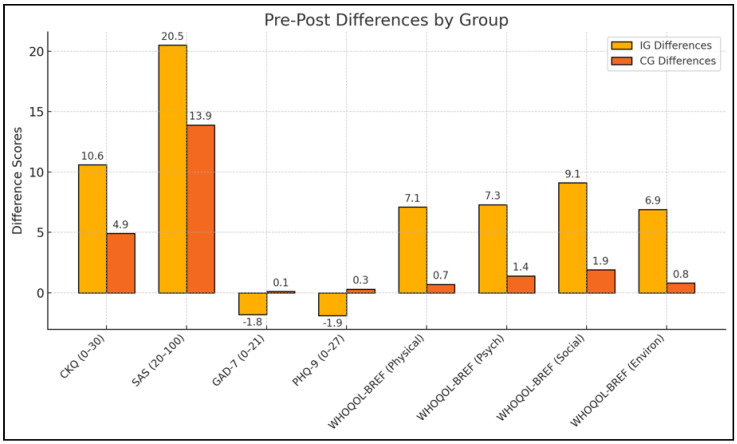
Pre-post differences by study group.

Following the 12-week intervention, the IG exhibited marked improvements on several key outcomes (Table 3 and Figure 1). The most pronounced change was observed in contraceptive knowledge (CKQ), which increased from a baseline mean of 14.9 to 25.5, whereas the CG also showed an increase but to a lesser extent (15.2 to 20.1). This difference was reflected in a large effect size (partial η^2^ = 0.20), indicating a robust impact of the counseling materials on knowledge acquisition. Improvements in sexual autonomy (SAS) were similarly greater in the IG, rising from 62.1 to 82.6 compared to a more moderate gain in the CG (61.8 to 75.7). Mental well-being measures further highlighted group disparities; while the IG demonstrated significant reductions in anxiety (GAD-7) and depression (PHQ-9), the CG experienced minimal changes or nonsignificant increases, suggesting that the structured counseling program may confer mental health benefits beyond enhancing contraceptive literacy.

Post-intervention comparisons revealed that participants in the IG demonstrated more pronounced improvements across several key outcomes. For instance, mean CKQ scores increased from 14.9 ± 3.2 at baseline to 25.5 ± 3.1 (*p* < 0.001) in the IG, compared with a more moderate rise in the CG (15.2 ± 3.0 to 20.1 ± 4.3, *p* < 0.001). The effect of group membership on post-intervention CKQ remained statistically significant (F = 42.75, *p* < 0.001, partial η^2^ = 0.20) when adjusting for baseline CKQ, age, and relationship status (Table 4), suggesting that the structured contraceptive counseling program exerted a robust influence on knowledge acquisition.

Sexual autonomy (SAS) also improved more substantially in the IG (62.1 ± 10.3 to 82.6 ± 9.2, *p* < 0.001) than in the CG (61.8 ± 9.9 to 75.7 ± 10.1, *p* < 0.001), reflecting a medium effect size (partial η^2^ = 0.11) and indicating that enhanced reproductive health education may bolster women’s decision-making confidence. Concomitantly, mental health indicators diverged notably between the two groups; IG participants showed meaningful reductions in anxiety (GAD-7: 4.9 ± 2.1 to 3.1 ± 2.0, *p* < 0.001) and depression (PHQ-9: 6.7 ± 2.2 to 4.8 ± 2.0, *p* < 0.001), whereas the CG exhibited either negligible or nonsignificant changes on these measures (e.g., GAD-7: 5.1 ± 2.3 to 5.2 ± 2.3, *p* = 0.481). These differences persisted when controlling for relevant covariates, underscoring the potential mental health benefits linked to improved contraceptive knowledge and skills.

In terms of overall quality of life (WHOQOL-BREF), the IG again outperformed the CG in each domain (physical, psychological, social, and environmental) at follow-up. The social domain displayed one of the largest between-group effects (F = 23.55, *p* < 0.001, partial η^2^ = 0.17), with IG scores increasing from 67.8 ± 14.3 to 76.9 ± 13.2 (*p* < 0.001) versus a smaller, nonsignificant rise in the CG (66.1 ± 13.8 to 68.0 ± 14.0, *p* = 0.212). These findings suggest that the intervention may have contributed not only to greater reproductive health literacy but also to enhanced psychosocial support and well-being.

**Figure 2:**
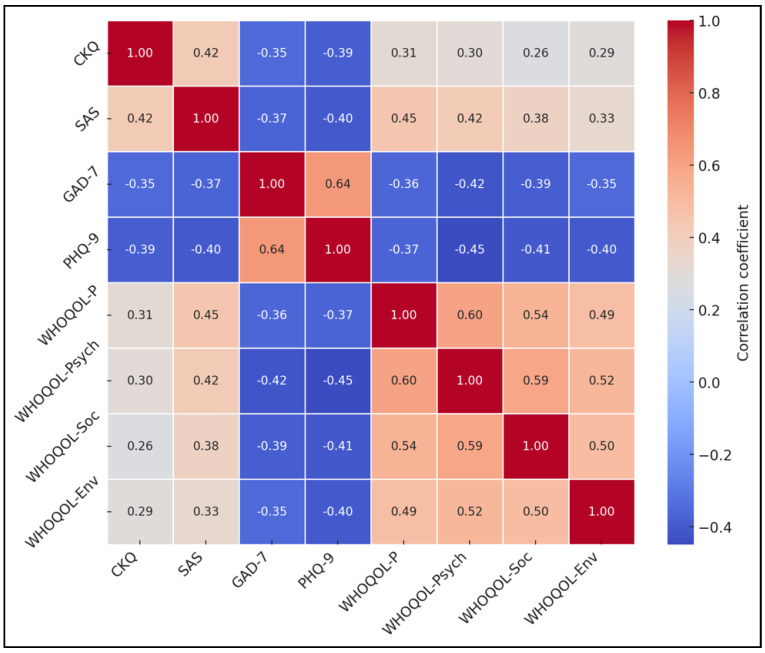
Correlation heatmap.

Correlation analyses provided further insight into the interplay among study variables. Higher CKQ and SAS scores were positively associated with WHOQOL-BREF physical, psychological, social, and environmental domains (r values from 0.26 to 0.45, all *p* < 0.001) and inversely correlated with GAD-7 and PHQ-9 scores (r values from −0.35 to −0.40, *p* < 0.001). Conversely, GAD-7 and PHQ-9 were strongly correlated with each other (r = 0.64, *p* < 0.001), indicating a shared variance in anxiety and depressive symptoms, as seen in Table 5, Figure 2.

The model (R^2^ = 0.42, F = 28.3, *p* < 0.001) shows a constant of 8.1 (*p* < 0.001). Group membership (IG vs. CG) had a coefficient of 5.2 (*p* < 0.001), and baseline CKQ had a coefficient of 0.5 (*p* < 0.001). Relationship status had a coefficient of 1.1 (*p* = 0.103), and age had a coefficient of −0.1 (*p* = 0.364) (Table 6).

The model (R^2^ = 0.38, F = 22.0, *p* < 0.001) includes a constant of 6.1 (*p* < 0.001). Group membership (IG vs. CG) had a coefficient of −2.8 (*p* < 0.001), while baseline PHQ-9 showed a coefficient of 0.45 (*p* < 0.001). Relationship status had a coefficient of −0.6 (*p* = 0.297), and age had a coefficient of 0.1 (*p* = 0.213) (Table 7).

## 4. Discussion

### 4.1. Important Findings and Literature Review

Offering contraceptive knowledge materials yielded substantial improvements in contraceptive knowledge and sexual autonomy, concomitant with better mental well-being among participants. Notably, even though both groups showed some gains (the CG possibly benefited from minimal resources provided and general academic exposure), the magnitude of positive change was significantly higher in the IG. This finding underscores the importance of targeted interventions rather than relying on passive informational materials.

The findings from the PHQ-9 and GAD-7 assessments reveal a troubling trend of higher depression and anxiety levels among future mothers, reinforcing the notion that having children during academic pursuits may exacerbate mental health challenges. This correlation could be influenced by several factors, including financial pressures, social isolation, and the stigmatization of future young mothers. Addressing these mental health disparities requires a holistic approach that integrates mental health services with academic and social support, emphasizing the importance of accessible mental health resources tailored to the needs of future parents. The stark differences in contraceptive knowledge and its perceived impact on life decisions between the two groups demonstrate the pivotal role of effective contraceptive education in empowering women to make informed choices.

In a similar manner, the study by Lemma et al. [23] found that contraceptive decision making among young college students in Ethiopia emphasized the role of relationship dynamics, communication, and mutual trust in determining contraceptive use. Their findings revealed that participants in intimate and long-term relationships engaged in open discussions about contraception and were more likely to use contraceptives consistently. In contrast, those in casual relationships displayed lower levels of communication and trust, often lacking commitment to shared contraceptive decision making. Similarly, Kara et al. [24] conducted a study in Tanzania to assess knowledge, attitude, and practice of contraception among female undergraduates, uncovering high awareness levels of contraception (96% of participants) but a moderate actual usage rate (47.4%). Factors such as embarrassment in purchasing contraceptives and religious beliefs significantly impacted contraceptive use.

It is worth mentioning that the current study took place at the end of the COVID-19 pandemic, that could have influenced the contraceptive practices. In a similar manner, the study by Chen et al. [25] investigated contraceptive access among students in North Carolina during the COVID-19 pandemic, revealing that 95% of respondents maintained access to their preferred methods despite campus closures. This study highlighted that students in longer degree programs experienced less disruption compared to those in 2-year colleges, and nonbinary and transgender students were more likely to lose access, indicating significant disparities.

In this field, although we used in our study four generally validated scales, other more specific surveys were recently developed, such as the one by Sanz-Martos et al. [26], who developed a validated instrument to measure knowledge about sexuality and contraception, finding it reliable and effective in distinguishing levels of knowledge among students. The research team constructed a 15-item instrument. Their findings demonstrated that the instrument was not only reliable—with a high reliability coefficient of 0.99 for items and 0.74 for respondents—but also exhibited good temporal stability, with a test-retest reliability score of 0.81.

As Romania still struggles with teenage pregnancies and high rates of unplanned pregnancies in young adults compared to the average in the European Union, it is necessary to understand sexual education, contraception knowledge, and sexual practices, and the implications that these have on the lives of the affected individuals. Regarding other lower-income countries, it was observed that in Colombia, 52.3% of students possessed adequate contraceptive knowledge, and 80.1% exhibited positive attitudes, with effective sex education and parental discussions identified as significant enhancers [27]. Conversely, another study from India [28] showed a high awareness of contraceptives (98% aware of family planning; 86% knew about contraceptives), yet actual usage remained low, with only 11% having used contraceptives. These findings underscore the gap between knowledge and practice, influenced by socio-economic, educational, and cultural barriers, highlighting the need for tailored educational strategies that not only raise awareness but also encourage practical application and acceptance of contraceptive methods to prevent unwanted pregnancies and support responsible sexual behaviors.

Nevertheless, the results of this study reveal that having children negatively impacts the quality of life and mental health of female college students in Romania, aligning with existing literature that highlights the multifaceted challenges faced by student parents [29]. Beyond the dual stressors of academic responsibilities and parenthood, economic constraints and inadequate social support systems likely exacerbate these negative outcomes [30]. The significant negative correlations between the number of children and quality of life measures, as well as the positive correlations with depression and anxiety, suggest that additional familial responsibilities may strain students’ ability to maintain their academic performance and personal well-being.

For a more comprehensive understanding, it is essential to explore additional factors that may contribute to these findings. Beyond the dual stressors of academic responsibilities and parenthood, economic challenges and a lack of social support systems likely play significant roles in exacerbating these negative outcomes [31]. Furthermore, the elevated levels of depression and anxiety observed among students with children could be intricately linked to social stigma and the increased academic workload they face. In many cultural contexts, young mothers may encounter societal judgment and limited acceptance, which can intensify their mental health struggles and hinder their academic performance [32]. To address these issues effectively, the study not only recommends enhancing contraceptive education but also suggests the implementation of specific policy measures. For instance, introducing comprehensive support programs similar to those successfully implemented in Scandinavian countries could provide the necessary framework to support young mothers academically and emotionally [33]. Additionally, policymakers and educational institutions should consider developing targeted interventions that include financial assistance, accessible childcare services, and robust mental health support tailored to the unique needs of student parents. By incorporating these strategies, the findings of this study can be translated into actionable policies that significantly improve the well-being and academic success of college women with children.

It is also important to recognize that the CKQ was devised to cater specifically to the nuances of contraceptive knowledge pertinent to Romanian college women. While content validity was supported through consultations with experts in reproductive health, comprehensive validation measures such as internal consistency and test-retest reliability were not initially reported. This oversight could potentially impact the strength and interpretability of the conclusions drawn regarding contraceptive knowledge. Ongoing efforts to further validate the CKQ are essential and will provide a more rigorous methodological foundation for interpreting the impact of contraceptive education interventions.

### 4.2. Study Limitations

This study, while informative, has several limitations that must be acknowledged. The reliance on self-reported data could introduce response bias, potentially affecting the accuracy of the reported mental health statuses and contraceptive knowledge. Additionally, the sample was restricted to a single university, which might limit the generalizability of the findings to other populations, and the estimated sample size was not met entirely for the group of female students with children. Furthermore, the study did not account for all possible confounding variables, such as socioeconomic status or educational level of the students, which might influence both the contraceptive knowledge, decision to have children and the reported outcomes.

## 5. Conclusions

This study conclusively demonstrates that a structured contraceptive counseling program substantially boosts reproductive health knowledge, sexual autonomy, and mental well-being among Romanian college women, aligning with our initial objectives as detailed in the abstract. Specifically, the program participants showed marked improvements in contraceptive knowledge and reported lower levels of anxiety and depression, compared to the control group. These outcomes suggest that university health services could significantly benefit from integrating systematic, culturally informed contraceptive education. Future research should explore the long-term impacts of such programs and investigate their applicability in different cultural settings. This approach ensures a clear distinction from the broader discussions in the previous sections and focuses on directly addressing the aims and implications of our findings.

## Figures and Tables

**Table 1:** Baseline demographic characteristics of participants.

Variable	IG (n = 115)	CG (n = 116)	*p*-Value
Age, years (mean ± SD)	20.8 ± 1.7	21.0 ± 1.9	0.432
BMI, kg/m^2^ (mean ± SD)	22.4 ± 2.9	22.1 ± 3.1	0.531
Place of Origin (Urban)	74 (64.3%)	76 (65.5%)	0.856
Relationship Status (Yes)	69 (60.0%)	68 (58.6%)	0.833
Smoking (Yes)	22 (19.1%)	19 (16.4%)	0.57
Alcohol Use (At least monthly)	48 (41.7%)	51 (44.0%)	0.687
Past STD Diagnosis (Yes)	9 (7.8%)	5 (4.3%)	0.247

SD—standard deviation; BMI—body mass index; STD—sexually transmitted disease.

**Table 2:** Baseline scores for primary study measures.

Measure	IG (n = 115)	CG (n = 116)	*p*-Value
CKQ (0–30)	14.9 ± 3.2	15.2 ± 3.0	0.514
SAS (20–100)	62.1 ± 10.3	61.8 ± 9.9	0.81
WHOQOL-BREF (Physical)	69.2 ± 13.4	70.5 ± 12.8	0.497
WHOQOL-BREF (Psych)	65.5 ± 11.1	64.8 ± 12.0	0.666
WHOQOL-BREF (Social)	67.8 ± 14.3	66.1 ± 13.8	0.39
WHOQOL-BREF (Environ)	70.2 ± 11.5	71.0 ± 10.8	0.608
GAD-7 (0–21)	4.9 ± 2.1	5.1 ± 2.3	0.522
PHQ-9 (0–27)	6.7 ± 2.2	6.8 ± 2.1	0.721

GAD—general anxiety disorder (higher scores indicate higher anxiety symptoms); PHQ—patient health questionnaire (higher scores indicate more severe depression symptoms).

**Table 3:** Pre-post comparisons by group.

Measure	IG Baseline	IG Post	*p* (Within IG)	CG Baseline	CG Post	*p* (Within CG)
CKQ (0–30)	14.9 ± 3.2	25.5 ± 3.1	<0.001	15.2 ± 3.0	20.1 ± 4.3	<0.001
SAS (20–100)	62.1 ± 10.3	82.6 ± 9.2	<0.001	61.8 ± 9.9	75.7 ± 10.1	<0.001
GAD-7 (0–21)	4.9 ± 2.1	3.1 ± 2.0	<0.001	5.1 ± 2.3	5.2 ± 2.3	0.481
PHQ-9 (0–27)	6.7 ± 2.2	4.8 ± 2.0	<0.001	6.8 ± 2.1	7.1 ± 2.2	0.208
WHOQOL-BREF (Physical)	69.2 ± 13.4	76.3 ± 11.5	<0.001	70.5 ± 12.8	71.2 ± 13.1	0.428
WHOQOL-BREF (Psych)	65.5 ± 11.1	72.8 ± 10.4	<0.001	64.8 ± 12.0	66.2 ± 11.7	0.239
WHOQOL-BREF (Social)	67.8 ± 14.3	76.9 ± 13.2	<0.001	66.1 ± 13.8	68.0 ± 14.0	0.212
WHOQOL-BREF (Environ)	70.2 ± 11.5	77.1 ± 10.1	<0.001	71.0 ± 10.8	71.8 ± 10.9	0.402

WHOQOL-BREF—Brief Version of the World Health Organization Quality of Life survey (higher scores indicate better quality of life).

**Table 4:** Adjusted post-intervention group differences (ANCOVA).

Measure	IG (Mean ± SE)	CG (Mean ± SE)	F	*p*-Value	Partial η^2^
CKQ	25.7 ± 0.6	20.3 ± 0.6	42.75	<0.001	0.2
SAS	82.4 ± 1.7	76.1 ± 1.7	12.91	0.001	0.11
GAD-7	3.2 ± 0.3	5.1 ± 0.3	16.02	<0.001	0.13
PHQ-9	4.9 ± 0.3	7.0 ± 0.3	18.64	<0.001	0.14
WHOQOL-P	76.5 ± 1.2	71.5 ± 1.2	9.21	0.003	0.08
WHOQOL-Psych	73.2 ± 1.1	66.6 ± 1.1	18.7	<0.001	0.14
WHOQOL-Soc	77.3 ± 1.3	68.8 ± 1.3	23.55	<0.001	0.17
WHOQOL-Env	77.6 ± 1.1	72.1 ± 1.1	14.39	<0.001	0.12

CKQ, Contraceptive Knowledge Questionnaire; SAS, Sexual Autonomy Scale; GAD-7, Generalized Anxiety Disorder-7; PHQ-9, Patient Health Questionnaire-9; WHOQOL-P, WHOQOL-BREF physical domain; WHOQOL-Psych, psychological domain; WHOQOL-Soc, social domain; WHOQOL-Env, environmental domain.

**Table 5:** Pearson’s correlation matrix.

	CKQ	SAS	GAD-7	PHQ-9	WHOQOL-P	WHOQOL-Psych	WHOQOL-Soc	WHOQOL-Env
CKQ	1	0.42 *	−0.35 *	−0.39 *	0.31 *	0.30 *	0.26 *	0.29 *
SAS	0.42 *	1	−0.37 *	−0.40 *	0.45 *	0.42 *	0.38 *	0.33 *
GAD-7	−0.35 *	−0.37 *	1	0.64 *	−0.36 *	−0.42 *	−0.39 *	−0.35 *
PHQ-9	−0.39 *	−0.40 *	0.64 *	1	−0.37 *	−0.45 *	−0.41 *	−0.40 *
WHOQOL-P	0.31 *	0.45 *	−0.36 *	−0.37 *	1	0.60 *	0.54 *	0.49 *
WHOQOL-Psych	0.30 *	0.42 *	−0.42 *	−0.45 *	0.60 *	1	0.59 *	0.52 *
WHOQOL-Soc	0.26 *	0.38 *	−0.39 *	−0.41 *	0.54 *	0.59 *	1	0.50 *
WHOQOL-Env	0.29 *	0.33 *	−0.35 *	−0.40 *	0.49 *	0.52 *	0.50 *	1

* All correlations significant at *p* < 0.001.

**Table 6:** Multiple linear regression predicting post-intervention CKQ scores.

Predictor	B	SE	β	t	*p*
(Intercept)	8.1	2.1	–	3.86	<0.001
Group (IG vs. CG)	5.2	0.82	0.48	6.34	<0.001
Baseline CKQ	0.5	0.09	0.39	5.56	<0.001
Relationship Status	1.1	0.67	0.1	1.64	0.103
Age	−0.1	0.11	−0.05	−0.91	0.364
Model Summary			R^2^ = 0.42	F = 28.3	<0.001

**Table 7:** Multiple linear regression predicting post-intervention PHQ-9 scores.

Predictor	B	SE	β	t	*p*
(Intercept)	6.1	1.3	–	4.69	<0.001
Group (IG vs. CG)	−2.8	0.57	−0.33	−4.91	<0.001
Baseline PHQ-9	0.45	0.1	0.31	4.5	<0.001
Relationship Status	−0.6	0.57	−0.07	−1.05	0.297
Age	0.1	0.08	0.07	1.25	0.213
Model Summary			R^2^ = 0.38	F = 22.0	<0.001

## Data Availability

The data presented in this study are available on request from the corresponding author.
